# Supplementary material for: The causal relationship between the human gut microbiota and pyogenic arthritis: a Mendelian randomization study
Source: Front Cell Infect Microbiol. 2024 Nov 26;14:1452480. doi: 10.3389/fcimb.2024.1452480 (PMC11629706; doi:10.3389/fcimb.2024.1452480)
Supplement: Supplementary file 1 [file DataSheet1.zip › mendelian test/ebi-a-GCST90027772.csv_leave_one_out.pdf]

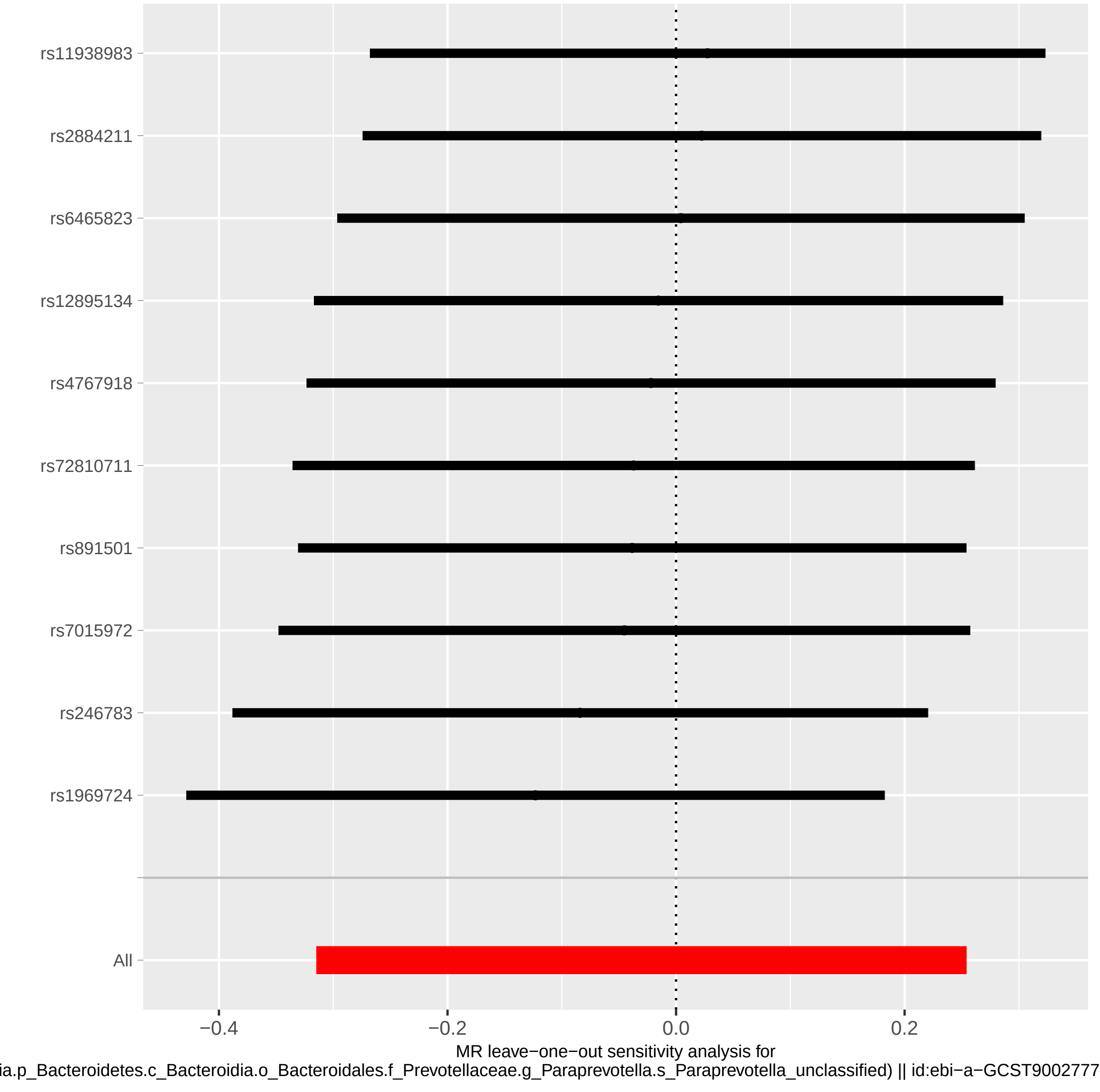
rs11938983
rs2884211
rs6465823
rs12895134
rs4767918
rs72810711
rs891501
rs7015972
rs246783
rs1969724
All
−0.4
−0.2
0.0
0.2
MR leave−one−out sensitivity analysis for
ia.p_Bacteroidetes.c_Bacteroidia.o_Bacteroidales.f_Prevotellaceae.g_Paraprevotella.s_Paraprevotella_unclassified) || id:ebi−a−GCST9002777
